# Excessive Daytime Sleepiness in Hypertensive Patients: The Role of Major Depressive Disorder

**DOI:** 10.3390/diagnostics14171854

**Published:** 2024-08-24

**Authors:** Alexandre Younes, Camille Point, Benjamin Wacquier, Jean-Pol Lanquart, Matthieu Hein

**Affiliations:** 1 Faculté de Médecine, Université Libre de Bruxelles, ULB, 1070 Bruxelles, Belgium; alexandre.younes@ulb.be; 2 Hôpital Universitaire de Bruxelles, Service de Psychiatrie et Laboratoire du Sommeil, Université Libre de Bruxelles, ULB, 1070 Bruxelles, Belgium; camille.point@hubruxelles.be (C.P.); secmed.psy@erasme.ulb.ac.be (J.-P.L.); 3 Laboratoire de Psychologie Médicale et Addictologie (ULB312), Université Libre de Bruxelles, ULB, 1020 Bruxelles, Belgium

**Keywords:** excessive daytime sleepiness, major depressive disorder, hypertension, sleep disorders, polysomnography

## Abstract

There is a special relationship between major depressive disorder and excessive daytime sleepiness. However, given the negative impact of excessive daytime sleepiness on life quality and cardiovascular outcome in hypertensive patients, the objective of this study was to investigate the potential role played by major depressive disorder in the occurrence of this complaint for this particular subpopulation. Data from 1404 hypertensive patients recruited from the Sleep Unit’s polysomnographic recordings database were analyzed. A score >10 on the Epworth Sleepiness Scale was used to define excessive daytime sleepiness in this study. Logistic regression analyses were performed to investigate the risk of excessive daytime sleepiness associated with major depressive disorder in hypertensive patients. Excessive daytime sleepiness was frequent (40.0%) in our sample of hypertensive patients. After adjustments for major confounding factors, multivariate logistic regression analyses demonstrated that unlike remitted major depressive disorder, only current major depressive disorder was associated with a higher risk of excessive daytime sleepiness in hypertensive patients. Given this potential implication of current major depressive disorder in the occurrence of excessive daytime sleepiness for hypertensive patients, it is therefore essential to achieve the complete remission of this psychiatric disorder to avoid negative consequences associated with this complaint in this particular subpopulation.

## 1. Introduction

Excessive daytime sleepiness is an important public health problem since it is a frequent complaint in the general population (23.3%) associated with multiple deleterious consequences (reduced life quality, poorer occupational outcome, an increased risk of car accidents and higher cardiovascular mortality) [1,2,3,4,5,6,7]. However, based on the few studies available in the literature, the prevalence of excessive daytime sleepiness seems to be higher (36.0 to 62.8%) in hypertensive patients than in the general population [8,9,10]. Additionally, there appears to be a bidirectional relationship between excessive daytime sleepiness and hypertension since excessive daytime sleepiness is associated with a higher risk of hypertension and some antihypertensive medications may induce excessive daytime sleepiness [11,12]. Furthermore, in hypertensive patients, the co-occurrence of excessive daytime sleepiness may be associated with reduced life quality, poorer long-term adherence to anti-hypertensive medications and a less favorable cardiovascular outcome [13,14,15]. Thus, given this potential negative impact on life quality and cardiovascular outcome, it seems essential to identify the risk factors for excessive daytime sleepiness in hypertensive patients to allow a better understanding of the frequent occurrence of this complaint in this particular subpopulation.

In the literature, there are arguments for a particular relationship between major depressive disorder and excessive daytime sleepiness. Indeed, major depressive disorder is associated with a higher risk of excessive daytime sleepiness whereas excessive daytime sleepiness may promote the development of major depressive disorder [16,17,18]. Furthermore, the prevalence of major depressive disorder is estimated at 9.2% in individuals with severe daytime sleepiness whereas excessive daytime sleepiness may affect 50.8% of major depressed individuals [19,20]. Despite the existence of this special relationship between excessive daytime sleepiness and major depressive disorder, the involvement of major depressive disorder in the occurrence of excessive daytime sleepiness has not been investigated in hypertensive patients. However, in this specific subpopulation, the occurrence of major depressive disorder seems to be more frequent (26.8%) than in the general population (9.2%), which could be explained by the use of some anti-hypertensive medications (beta-blockers or calcium antagonists) and/or by the negative impact of chronic pathologies on psychological functioning [21,22,23,24]. Given these different elements, there is a strong interest in the study of the risk of excessive daytime sleepiness associated with major depressive disorder in hypertensive patients in order to open new perspectives for the management of this complaint in this particular subpopulation.

The first objective of this study was to investigate the prevalence of excessive daytime sleepiness in a large sample of hypertensive patients. The second objective of this study was to demonstrate that independently of the classic risk factors [25,26], major depressive disorder is associated with a higher risk of excessive daytime sleepiness in hypertensive patients. The aim of this approach is to provide healthcare professionals with new perspectives for the management of excessive daytime sleepiness in hypertensive patients in order to improve their life quality and their cardiovascular outcome.

## 2. Materials and Methods

### 2.1. Recruitment of Hypertensive Patients

In total, 1404 hypertensive patients who underwent a polysomnography between 1 January 2002 and 31 December 2020 were retrospectively selected from the database of the Sleep Unit of the Brussels University Hospital. For this selection of hypertensive patients, the inclusion criteria applied were as follows: (1) age ≥ 18 years; (2) presence of hypertension according to the diagnostic criteria of the World Health Organization (mean systolic blood pressure ≥ 140 mmHg or mean diastolic blood pressure ≥ 90 mmHg or self-reported diagnosis of medically validated hypertension or use of antihypertensive medication) [27,28,29]; (3) absence of psychiatric disorders other than major depressive disorder; (4) absence of substance use disorders and (5) absence of pregnancy. The exclusion criteria applied were as follows: (1) presence of acute and/or uncontrolled medical/inflammatory/infectious pathologies, (2) presence of congenital and/or acquired brain lesions, (3) presence of craniofacial and/or thoracic malformations, (4) history of head trauma, (5) presence of some sleep disorders (central hypersomnia or predominantly central sleep apnea syndrome) and (6) presence of obstructive sleep apnea syndrome already known or treated before admission to the Sleep Unit. In this study, recruitment focused only on hypertensive patients since the aim was to specifically target this particular subpopulation in which excessive daytime sleepiness appears to be associated with a negative impact on life quality, long-term adherence to anti-hypertensive medications and cardiovascular outcome [13,14,15]. Furthermore, this study was approved by the Ethics Committee of Erasme Hospital (Reference: P2023/481—Submission date: 20 November 2023—Approval date: 12 January 2024). Finally, the Supplementary Data—Annex S1 contains the description of the care pathway for these hypertensive patients from the first outpatient consultation specialized in sleep medicine until admission to the Sleep Unit of the Brussels University Hospital.

### 2.2. Methods

#### 2.2.1. Medical Check-Up and Psychiatric Interview of Hypertensive Patients

Upon admission of all these hypertensive patients, a physician assigned to the Sleep Unit performed a review of medical records, a clinical interview, a physical examination and complementary tests (electrocardiogram, daytime electroencephalogram, blood test and urine analysis) to allow a complete medical check-up and diagnose potential somatic comorbidities. In addition, during this complete medical check-up, a control of potential antihypertensive medications and repeated blood pressure measurements (Supplementary Data—Annex S2) were carried out by the medical and nursing staff of the Sleep Unit to confirm the diagnosis of hypertension highlighted during the outpatient assessment and define the status of hypertension (untreated, controlled [adequate response to antihypertensive medications with mean systolic blood pressure < 140 mmHg and mean diastolic blood pressure < 90 mmHg] and uncontrolled [inadequate response to antihypertensive medications with mean systolic blood pressure ≥ 140 mmHg and/or mean diastolic blood pressure ≥ 90 mmHg]) [27,28,29].

After this complete medical check-up, a psychiatrist assigned to the Sleep Unit performed a systematic psychiatric interview based on the diagnostic criteria of DSM-IV-TR (before 2013) or DSM 5 (after 2013) in order to screen for potential current or past comorbid psychiatric disorders in all these hypertensive patients [30,31]. Furthermore, in case of current or past major depressive disorder highlighted during this systematic psychiatric interview, the status of major depressive episodes was defined based on the following criteria:

- Absence of significant symptoms or signs of major depressive disorder for a period of at least 2 months before admission to the Sleep Unit for remitted major depressive episodes [30,31].
- Presence of significant symptoms or signs of major depressive disorder according to the diagnostic criteria of DSM IV-TR (before 2013) or DSM 5 (after 2013) for a period of at least 2 weeks before admission to the Sleep Unit for current major depressive episodes [30,31].

Finally, in all these hypertensive patients, an assessment of their subjective complaints of depression, insomnia and daytime sleepiness was carried out using self-questionnaires (Beck Depression Inventory reduced to 13 items [BDI-II], Insomnia Severity Index and Epworth Sleepiness Scale) (Supplementary Data—Annex S3) [32,33,34].

#### 2.2.2. Sleep Interview and Polysomnographic Recording of Hypertensive Patients

After this medical and psychiatric assessment, a semi-structured interview specific to the Sleep Unit of the Brussels University Hospital was carried out by a physician specializing in sleep medicine in all these hypertensive patients to assess their sleep habits and research for the presence of potential clinical manifestations (signs and/or symptoms) of comorbid sleep disorders. In addition, all these hypertensive patients benefited from a polysomnographic recording characterized by a montage meeting the technical criteria of the American Academy of Sleep Medicine and performed under the usual hospitalization conditions of the Sleep Unit (Supplementary Data—Annex S4) [35]. Afterwards, based on the scoring criteria of the American Academy of Sleep Medicine (Supplementary Data—Annex S5) [36,37,38], all of these polysomnographic recordings were visually analyzed by technicians supervised by certified somnologists in order to allow the production of technical reports integrated into the medical records of all these hypertensive patients. So, thanks to this semi-structured sleep interview and this polysomnographic recording, all hypertensive patients selected for this study benefited from systematic screening for their potential comorbid sleep disorders: obstructive sleep apnea syndrome (absent [obstructive apnea-hypopnea index < 5/h], mild [obstructive apnea-hypopnea index 5–14/h], moderate-to-severe [obstructive apnea-hypopnea index ≥ 15/h]), insomnia disorder, short sleep duration (<6 h), restless legs syndrome and moderate-to-severe periodic limb movement syndrome (periodic limb movement index ≥ 15/h) [39,40,41,42,43].

### 2.3. Statistical Analyses

Stata software version 14 was used to carry out the statistical analyses for this study. During these analyses, hypertensive patients with an Epworth Sleepiness Scale score ≤ 10 were included in the group without excessive daytime sleepiness whereas those with an Epworth Sleepiness Scale score > 10 were included in the group with excessive daytime sleepiness [34].

To determine the statistical tests to apply for continuous data in this study, a verification of the conditions for applying parametric tests was carried out with a check of the normality of the distribution (histograms, boxplots and quantile–quantile plots) and equality of variances (Levene’s test). However, given that most of the continuous data in this study did not meet the conditions for using parametric tests, their descriptive analyses were performed using medians (P25–P75) and their comparison analyses between the 2 groups of hypertensive patients were performed using Wilcoxon tests (non-parametric tests). For categorical data, descriptive analyses were carried out using percentages and the comparison analyses between the 2 groups of hypertensive patients were carried out using Chi^2^ tests. Finally, Spearman correlation analyses with Bonferroni corrections for multiple analyses were used to investigate significant associations between the Epworth Sleepiness Scale total score and polysomnographic/clinical parameters.

The risk of excessive daytime sleepiness associated with major depressive disorder (categorized: no, remitted, current) and potential confounding factors (Supplementary Data—Annex S6) was investigated using univariate logistic regression analyses. Subsequently, the significant confounding factors identified during these univariate logistic regression analyses were introduced hierarchically using multivariate logistic regression analyses in order to adjust the risk of excessive daytime sleepiness associated with major depressive disorder. Finally, after this adjustment through the hierarchical introduction of these confounding factors significantly associated with excessive daytime sleepiness during univariate analyses, the adequacy and specificity of the final model obtained during these multivariate analyses were checked, respectively, using the Hosmer and Lemeshow test and the Link Test.

A *p*-value < 0.05 was considered significant.

## 3. Results

### 3.1. Sample Description (Table 1)

A description of the main clinical characteristics of the sample of hypertensive patients from this study is available in Table 1. Moreover, excessive daytime sleepiness was present in 40.0% of hypertensive patients from our sample. Finally, the prevalence of remitted and current major depressive disorder was 21.0% and 23.2%, respectively, in our sample of hypertensive patients.

**Table 1:** Sample description (*n* = 1404).

Variables	Median (P25–P75)	
Age (years)	52 (45–60)	
BMI (kg/m^2^)	29.6 (26.2–33.8)	
CRP (mg/L)	1.9 (1.0–3.8)	
Glycaemia (mg/dL)	94 (86–105)	
Total cholesterol (mg/dL)	191 (167–217)	
Triglyceride (mg/dL)	131 (93–188)	
HDL cholesterol (mg/dL)	50 (41–61)	
ESS	9 (6–13)	
ISI	14 (9–18)	
BDI	4 (2–8)	
Number of antihypertensive treatments	1 (0–2)	
Systolic blood pressure (mmHg)	138 (120–140)	
Diastolic blood pressure (mmHg)	80 (70–90)	
	Categories	%
Gender	Female (*n* = 435) male (*n* = 969)	31.0% 69.0%
Age (years)	≥50 (*n* = 830) <50 (*n* = 574)	59.1% 40.9%
BMI (kg/m²)	<25 (*n* = 252) ≥25 & <30 (*n* = 486) ≥30 (*n* = 666)	17.9% 34.6% 47.5%
Antidepressant therapy	No (*n* = 1078) Yes (*n* = 326)	76.8% 23.2%
Anxiolytic medication	No (*n* = 1203) Yes (*n* = 201)	85.7% 14.3%
Hypnotic medication	No (*n* = 1329) Yes (*n* = 75)	94.7% 5.3%
Smoking	No (*n* = 1131) Yes (*n* = 273)	80.6% 19.4%
Alcohol	No (*n* = 887) Yes (*n* = 517)	63.2% 36.8%
Caffeine	No (*n* = 279) Yes (*n* = 1125)	19.9% 80.1%
Type 2 diabetes	No (*n* = 1106) Yes (*n* = 298)	78.8% 21.2%
Dyslipidemia	No (*n* = 526) Untreated (*n* = 488) Treated (*n* = 390)	37.5% 34.8% 27.7%
Hypertension status	Untreated (*n* = 533) Controlled (*n* = 527) Uncontrolled (*n* = 344)	38.0% 37.5% 24.5%
Number of antihypertensive medications	0 (*n* = 533) 1 (*n* = 510) 2 (*n* = 236) ≥3 (*n* = 125)	38.0% 36.3% 16.8% 8.9%
OSAS	No (*n* = 566) Mild (*n* = 362) Moderate-to-severe (*n* = 476)	40.3% 25.8% 33.9%
Insomnia disorder	No (*n* = 469) Sleep deprivation alone (*n* = 314) Insomnia without short sleep duration (*n* = 367) Insomnia with short sleep duration (*n* = 254)	33.4% 22.4% 26.1% 18.1%
Sleep movement disorders	No (*n* = 1112) Moderate-to-severe PLMs alone (*n* = 110) RLS alone or combined with PLMs (*n* = 182)	79.2% 7.8% 13.0%
CRP (mg/L)	<5 (*n* = 1173) ≥5 (*n* = 231)	83.5% 16.5%
Major depression	No (*n* = 783) Remitted (*n* = 295) Current (*n* = 326)	55.8% 21.0% 23.2%
EDS	No (*n* = 843) Yes (*n* = 561)	60.0% 40.0%

BMI = body mass index; CRP = C-Reactive Protein; ESS = Epworth Sleepiness Scale; ISI = Insomnia Severity Index; BDI = Beck Depression Inventory; OSAS = obstructive sleep apnea syndrome; PLMs = periodic limb movements during sleep; RLS = restless legs syndrome.

### 3.2. Polysomnographic Data (Table 2)

Compared to hypertensive patients without excessive daytime sleepiness, hypertensive patients with excessive daytime sleepiness display the following:

- A reduction in sleep latency and percentage of wake after sleep onset.
- An increase in sleep efficiency, total sleep time, micro-arousal index, obstructive apnea-hypopnea index, oxygen desaturation index and total time under 90% of O2 saturation.

The two groups of hypertensive patients did not significantly differ for sleep period time, percentage of stage 1, percentage of stage 2, percentage of stage 3, percentage of REM sleep, REM latency, number of awakenings and periodic limb movement index.

**Table 2:** Polysomnographic data (*n* = 1404).

	Whole Sample (*n* = 1404)	Subjects without EDS (*n* = 843)	Subjects with EDS (*n* = 561)	*p*-Value
Sleep latency (min)	26.3 (14.4–50.5)	27.0 (15.5–52.0)	25.0 (13.3–45.0)	0.045
Sleep efficiency (%)	76.6 (66.2–83.9)	75.3 (64.4–82.9)	78.5 (68.7–85.6)	<0.001
Sleep period time (min)	449.5 (408.5–482.8)	449.5 (405.5–483.0)	450.7 (411.5–482.5)	0.346
Total sleep time (min)	376.0 (326.4–416.5)	371.0 (321.0–414.0)	383.3 (333.0–422.5)	0.002
% stage 1	8.1 (5.4–11.5)	8.0 (5.4–11.2)	8.3 (5.5–12.0)	0.072
% stage 2	54.9 (47.2–61.4)	54.9 (47.1–61.2)	55.3 (47.5–61.9)	0.254
% stage 3	2.3 (0.1–7.9)	2.2 (0.1–7.3)	2.6 (0.1–8.2)	0.585
% REM sleep	15.5 (10.9–19.3)	15.4 (10.7–19.2)	15.5 (11.3–19.5)	0.263
REM latency (min)	85.0 (60.0–137.5)	85.5 (60.0–137.3)	84.5 (60.3–140.3)	0.811
% WASO	14.0 (8.4–22.6)	14.8 (8.7–23.6)	12.8 (7.8–21.1)	0.003
Number of awakenings	32 (22–47)	32 (22–47)	32 (21–48)	0.715
Micro-arousal index	13 (8–22)	13 (8–21)	13 (8–26)	0.038
Apnea-hypopnea index	7 (2–22)	6 (2–19)	8 (2–29)	0.004
Oxygen desaturation index	3 (1–11)	3 (1–9)	4 (1–15)	<0.001
Total time under 90% of SaO_2_ (min)	6.3 (0.3–49.8)	5.3 (0.3–38.0)	9.5 (0.5–72.0)	0.017
PLMs index	2 (0–11)	2 (0–12)	2 (0–10)	0.147
	Median (P25–P75)	Median (P25–P75)	Median (P25–P75)	Wilcoxon test

EDS = excessive daytime sleepiness; REM = rapid eye movement; WASO = wake after sleep onset; SaO_2_ = oxygen saturation; PLMs = periodic limb movements during sleep.

### 3.3. Univariate Analyses (Table 3)

Age, body mass index, hypnotic use, dyslipidemia, hypertension status, the number of antihypertensive medications, obstructive sleep apnea syndrome, insomnia disorder, CRP levels and major depressive disorder were significantly associated with the occurrence of excessive daytime sleepiness in hypertensive patients. In addition, the two groups of hypertensive patients did not significantly differ for sex, antidepressant use, anxiolytic use, smoking, alcohol consumption, caffeine consumption, type 2 diabetes and sleep movement disorders. Finally, compared to hypertensive patients without excessive daytime sleepiness, hypertensive patients with excessive daytime sleepiness had higher body mass index, CRP levels, triglyceride levels, Epworth Sleepiness Scale scores, Beck Depression Inventory scores and Insomnia Severity Index scores whereas they had a younger age and a lower number of antihypertensive medications.

**Table 3:** Univariate analyses (*n* = 1404).

Variables	Categories	Subjects without EDS (*n* = 843)	Subjects with EDS (*n* = 561)	*p*-Value Chi^2^	OR (CI 95%)	*p*-Value
Gender	Female Male	31.0% 69.0%	31.0% 69.0%	0.983	1 0.99 (0.79 to 1.26)	0.983
Age (years)	≥50 <50	63.0% 37.0%	53.3% 46.7%	<0.001	1 1.49 (1.20 to 1.85)	<0.001
BMI (kg/m^2^)	<25 ≥25 & <30 ≥30	21.2% 36.2% 42.6%	13.0% 32.3% 54.7%	<0.001	1 1.46 (1.05 to 2.02) 2.10 (1.53 to 2.86)	<0.001
Antidepressant therapy	No Yes	78.2% 21.8%	74.7% 25.3%	0.130	1 1.21 (0.94 to 1.56)	0.130
Anxiolytic medication	No Yes	85.2% 14.8%	86.5% 13.5%	0.502	1 0.90 (0.66 to 1.22)	0.502
Hypnotic medication	No Yes	93.6% 6.4%	96.3% 3.7%	0.030	1 0.57 (0.34 to 0.95)	0.032
Smoking	No Yes	80.9% 19.1%	80.0% 20.0%	0.688	1 1.06 (0.81 to 1.38)	0.688
Alcohol	No Yes	63.8% 36.2%	62.2% 37.8%	0.540	1 1.07 (0.86 to 1.34)	0.540
Caffeine	No Yes	21.0% 79.0%	18.2% 81.8%	0.195	1 1.20 (0.91 to 1.57)	0.196
Type 2 diabetes	No Yes	77.5% 22.5%	80.8% 19.2%	0.140	1 0.82 (0.63 to 1.07)	0.140
Dyslipidemia	No Untreated Treated	38.4% 31.2% 30.4%	36.0% 40.1% 23.9%	0.001	1 1.37 (1.07 to 1.76) 0.84 (0.64 to 1.10)	0.001
Hypertension status	Untreated Controlled Uncontrolled	35.7% 36.9% 27.4%	41.4% 38.5% 20.1%	0.006	1 0.90 (0.71 to 1.15) 0.63 (0.48 to 0.84)	0.006
Number of antihypertensive medications	0 1 2 ≥3	35.7% 36.2% 17.8% 10.3%	41.4% 36.5% 15.3% 6.8%	0.032	1 0.87 (0.68 to 1.12) 0.74 (0.54 to 1.02) 0.57 (0.37 to 0.86)	0.033
OSAS	No Mild Moderate-to-severe	41.9% 26.9% 31.2%	38.0% 24.1% 37.9%	0.032	1 0.99 (0.75 to 1.29) 1.34 (1.05 to 1.72)	0.032
Insomnia disorder	No Sleep deprivation alone Insomnia without short sleep duration Insomnia with short sleep duration	35.7% 26.6% 21.2% 16.5%	30.0% 16.0% 33.5% 20.5%	<0.001	1 0.72 (0.53 to 0.98) 1.88 (1.42 to 2.49) 1.48 (1.09 to 2.02)	<0.001
Sleep movement disorders	No Moderate-to-severe PLMs alone RLS alone or combined with PLMs	77.9% 8.2% 13.9%	81.1% 7.3% 11.6%	0.346	1 0.86 (0.57 to 1.29) 0.80 (0.58 to 1.11)	0.347
CRP (mg/L)	<5 ≥5	85.2% 14.8%	81.1% 18.9%	0.044	1 1.34 (1.01 to 1.78)	0.045
Major depression	No Remitted Current	58.4% 23.4% 18.2%	51.9% 17.5% 30.6%	<0.001	1 0.84 (0.63 to 1.12) 1.89 (1.45 to 2.45)	<0.001
		**Median** **(P25–P75)**	**Median** **(P25–P75)**	**Wilcoxon Test**		
Age (years)		54 (46–62)	51 (43–56)	<0.001		
BMI (kg/m^2^)		28.7 (25.5–33.1)	30.4 (27.1–34.9)	<0.001		
CRP (mg/L)		1.8 (1.0–3.6)	2.0 (1.0–4.2)	0.031		
Glycaemia (mg/dL)		93 (85–106)	94 (87–104)	0.114		
Total cholesterol (mg/dL)		191 (166–215)	191 (168–220)	0.288		
Triglyceride (mg/dL)		125 (93–180)	138 (92–196)	0.027		
HDL cholesterol (mg/dL)		50 (42–61)	49 (41–59)	0.086		
ESS		6 (4–8)	14 (12–16)	<0.001		
ISI		13 (7–17)	15 (11–18)	<0.001		
BDI		3 (1–7)	5 (2–10)	<0.001		
Number of antihypertensive treatments		1 (0–2)	1 (0–1)	0.009		
Systolic blood pressure (mmHg)		140 (120–140)	135 (120–140)	0.581		
Diastolic blood pressure (mmHg)		80 (70–90)	80 (70–90)	0.292		

EDS = excessive daytime sleepiness; BMI = body mass index; OSAS = obstructive sleep apnea syndrome; PLMs = periodic limb movements during sleep; RLS = restless legs syndrome; CRP = C-Reactive Protein; ESS = Epworth Sleepiness Scale; ISI = Insomnia Severity Index; BDI = Beck Depression Inventory.

### 3.4. Multivariate Analyses (Table 4)

After having hierarchically introduced the significant confounding factors identified during the univariate analyses, multivariate logistic regression analyses highlighted that unlike remitted major depressive disorder, only current major depressive disorder was associated with the occurrence of excessive daytime sleepiness in hypertensive patients.

**Table 4:** Multivariate analyses (*n* = 1404).

Variables	Model 1 OR Adjusted (CI 95%)	*p*-Value	Model 2 OR Adjusted (CI 95%)	*p*-Value	Model 3 OR Adjusted (CI 95%)	*p*-Value	Model 4 OR Adjusted (CI 95%)	*p*-Value
Major depression No Remitted Current	1 0.87 (0.65 to 1.15) 1.95 (1.49 to 2.54)	<0.001	1 0.88 (0.66 to 1.18) 1.92 (1.47 to 2.52)	<0.001	1 0.88 (0.65 to 1.19) 1.68 (1.26 to 2.24)	<0.001	1 0.88 (0.65 to 1.18) 1.67 (1.25 to 2.24)	<0.001

Model 1 = Model adjusted for age and BMI. Model 2 = Model adjusted for age, BMI, dyslipidemia, hypertension status and number of antihypertensive treatments. Model 3 = Model adjusted for age, BMI, dyslipidemia, hypertension status, number of antihypertensive medications, OSAS, insomnia disorder and hypnotics. Model 4 = Model adjusted for age, BMI, dyslipidemia, hypertension status, number of antihypertensive medications, OSAS, insomnia disorder, hypnotics and CRP levels. BMI = body mass index; OSAS = obstructive sleep apnea syndrome; CRP = C-Reactive Protein.

### 3.5. Correlation Analyses (Table 5)

In our sample of hypertensive patients, the Epworth Sleepiness Scale total score was positively correlated with sleep efficiency, the oxygen desaturation index, the body mass index, the Insomnia Severity Index total score and the Beck Depression Inventory total score, and negatively with age and hypnotic medication use.

**Table 5:** Correlation analyses (*n* = 1404).

Polysomnographic Variables	ESS Total Score	Clinical Variables	ESS Total Score
Sleep latency (min)	−0.063	Age (years)	−0.147 *
Sleep efficiency (%)	0.118 *	BMI (kg/m^2^)	0.168 *
Sleep period time (min)	0.037	ISI total score	0.207 *
Total sleep time (min)	0.091	BDI total score	0.223 *
% stage 1	0.074	Number of antihypertensive medications	−0.035
% stage 2	0.035	Systolic blood pressure (mmHg)	−0.003
% stage 3	−0.008	Diastolic blood pressure (mmHg)	0.025
% REM sleep	0.043	Antidepressant therapy	0.009
REM latency (min)	−0.030	Anxiolytic medication	−0.056
% WASO	−0.083	Hypnotic medication	−0.118 *
Number of awakenings	0.032	Smoking	0.008
Micro-arousal index	0.071	Alcohol	0.017
Apnea-hypopnea index	0.103	Caffeine	0.040
Oxygen desaturation index	0.120 *	CRP (mg/L)	0.093
Total time under 90% of SaO_2_ (min)	0.087	Glycaemia (mg/dL)	0.053
PLMs index	0.087	Total cholesterol (mg/dL)	0.020
/		Triglyceride (mg/dL)	0.081
/		HDL cholesterol (mg/dL)	−0.076

* *p*-value <0.05; ESS = Epworth sleepiness scale; REM = rapid eye movement; WASO = wake after sleep onset; SaO_2_ = oxygen saturation; PLMs = periodic limb movements during sleep; BMI = body mass index; ISI = Insomnia Severity Index; BDI = Beck Depression Inventory; CRP = C-Reactive Protein.

## 4. Discussion

In this study, we demonstrated that the prevalence of excessive daytime sleepiness was 40.0% in our sample of hypertensive patients, which confirms that this complaint is more frequent in this subpopulation than in the general population. This prevalence is lower than that of the study by Mbatchou Ngahane et al. (2015) (62.8%) [9]. First, this difference in prevalence could be explained by the exclusion of individuals under hypnotic treatments in this study unlike our study. Indeed, the application of this exclusion criterion may have favored an overestimation of excessive daytime sleepiness in this study since in individuals with this complaint, the appropriate use of hypnotic treatments may be associated with a reduction in excessive daytime sleepiness through an improvement in the quantity and quality of sleep [44,45,46]. Second, psychiatric disorders other than major depressive disorder were not an exclusion criterion in the study by Mbatchou Ngahane et al. (2015) [9], which may have induced a selection bias favoring the inclusion of individuals at high risk of excessive daytime sleepiness in this study. Indeed, only individuals with untreated psychiatric disorders or treated with non-sedative treatments were included in this study. However, untreated psychiatric disorders are frequently associated with the occurrence of excessive daytime sleepiness whereas monotherapy with non-sedative psychotropic treatments may induce or worsen excessive daytime sleepiness through alterations in sleep continuity [20,26,47,48,49,50]. Furthermore, the prevalence of excessive daytime sleepiness demonstrated in our study is similar to that demonstrated by Drager et al. (2010) (36.0%), Uchmanowicz et al. (2019) (39.0%), Willame et al. (2022) (39.9%) and Draelants et al. (2023) (38.7%) [8,13,51,52], which seems to confirm a relative consistency of our results with the currently available literature and the importance of this problem in hypertensive patients. Finally, in this particular subpopulation, excessive daytime sleepiness is associated with the occurrence of negative consequences in terms of long-term adherence to anti-hypertensive medications and cardiovascular outcome [13,14,15], which justifies the establishment of adequate management of this complaint in order to avoid this deleterious impact of excessive daytime sleepiness in hypertensive patients.

In our study, we demonstrated that the prevalence of sleepy hypertensive patients with current major depressive disorder, sleepy hypertensive patients without current major depressive disorder, non-sleepy hypertensive patients with current major depressive disorder and non-sleepy hypertensive patients without current major depressive disorder was, respectively, 12.3%, 27.7%, 10.9% and 49.1% in our sample. However, the prevalence of sleepy hypertensive patients with current major depressive disorder (12.3%) represents approximately a third of the total prevalence of sleepy hypertensive subjects (40.0%), which seems to confirm the need to systematically research for the presence of major depressive disorder in hypertensive patients with excessive daytime sleepiness. Furthermore, similar to data available for the general population [53,54], we demonstrated that the presence of current major depressive disorder was associated with a higher risk of excessive daytime sleepiness in hypertensive patients, which could be explained by some specific factors. First, in our study, hypertensive patients with excessive daytime sleepiness had higher self-reported depression scores than those without excessive daytime sleepiness. However, some studies have demonstrated that the occurrence of excessive daytime sleepiness could be a reflection of this greater severity of self-reported complaints of depression [20,26,55,56]. Second, the use of antidepressant therapy without associated hypnotic treatment was more frequent in hypertensive patients with excessive daytime sleepiness than in those without excessive daytime sleepiness. However, it has been shown that the monotherapy prescription of some antidepressant treatments could promote or worsen excessive daytime sleepiness [20,26,57,58]. Third, compared to hypertensive patients without excessive daytime sleepiness, the proportion of young subjects was greater in those with excessive daytime sleepiness. However, in these younger individuals, atypical major depressive disorder is generally more frequent and is characterized by more marked alterations in daytime functioning (such as severe excessive daytime sleepiness) [20,59,60,61]. Alongside this higher risk of excessive daytime sleepiness associated with current major depressive disorder, we demonstrated that remitted major depressive disorder was not associated with a higher risk of excessive daytime sleepiness in hypertensive patients, which seems to indicate that only current major depressive disorder represents a risk factor for the occurrence of excessive daytime sleepiness in this particular subpopulation. Thus, given these elements, it seems essential to target the complete remission of major depressive disorder through strict compliance with current recommendations for the diagnosis and treatment of this psychiatric disorder [62,63] in order to avoid the persistence of excessive daytime sleepiness in hypertensive patients.

Furthermore, alongside their potential side effects that may negatively impact daytime functioning [64], some antihypertensive medications (such as angiotensin antagonists, beta blockers and calcium channel blockers) may promote the occurrence of major depressive disorder or worsen its severity [65,66], which could indirectly induce the development of excessive daytime sleepiness in hypertensive patients. Thus, in order to limit the risk of occurrence of excessive daytime sleepiness in this particular subpopulation, it seems necessary to choose adequate antihypertensive medication for subjects at risk of major depressive disorder or with current major depressive disorder to avoid inducing the development of major depressive disorder or aggravating the severity of pre-existing major depressive disorder in this specific subgroup of hypertensive patients [67,68].

In the literature, it has been shown that some sleep disorders (such as insomnia disorders, obstructive sleep apnea syndrome and restless legs syndrome alone or combined to periodic limb movements) may play a central role in the occurrence of excessive daytime sleepiness [69], which justifies the systematic screening and treatment of these sleep disorders given their high prevalence in hypertensive patients [70]. However, the co-occurrence of major depressive disorder and these sleep disorders is frequent [71,72], which could favor the development of a synergistic effect between these different pathologies on some pathophysiological mechanisms involved in the occurrence of excessive daytime sleepiness (such as alterations of duration and/or continuity of sleep) [73]. Thus, in hypertensive patients, in the case of co-occurrence of current major depressive disorder and sleep disorders, it seems essential to perform a combined management of these pathologies in order to avoid the maintenance of pathophysiological mechanisms favoring the persistence of excessive daytime sleepiness and its deleterious consequences on cardiovascular outcome and life quality [74,75,76].

### Limitations

Since this study was retrospective, the data used were not verified directly with the hypertensive patients included, which justifies the conduct of future prospective studies to validate our findings. Furthermore, given that the Epworth Sleepiness Scale was used to assess daytime sleepiness in this study, only a subjective measure of this complaint was available for our analyses, which may potentially limit the generalization of our results following the absence of objective measurement (such as multiple sleep latency test). In addition, we only included hypertensive patients in this study, which means that our findings cannot be extrapolated to other cardiovascular pathologies. Finally, since the database of the Sleep Unit of the Brussels University Hospital only contains individuals who have agreed to undergo a polysomnographic recording, a recruitment bias may have occurred during the selection of hypertensive patients included in this study, which could limit the interpretation of our results.

## 5. Conclusions

In this study, we demonstrated that excessive daytime sleepiness is a frequent complaint (40.0%) in hypertensive patients. Furthermore, we highlighted that unlike remitted major depressive disorder, only current major depressive disorder was associated with a more frequent occurrence of this complaint in hypertensive patients, which highlights the importance of achieving the complete remission of this psychiatric disorder to avoid negative consequences associated with excessive daytime sleepiness in this particular subpopulation. Finally, future additional studies must be carried out to validate our findings and allow the establishment of new strategies for managing excessive daytime sleepiness in hypertensive patients.

## Data Availability

The data presented in this study are available on request from the corresponding author due to internal institutional procedures.

## Supplementary Materials

The following supporting information can be downloaded at: https://www.mdpi.com/article/10.3390/diagnostics14171854/s1, Annex S1: Outpatient care pathway for hypertensive patients; Annex S2: Description of blood pressure measurement by nursing staff; Annex S3: Description of Beck Depression Inventory reduced to 13 items, Insomnia Severity Index and Epworth Sleepiness Scale; Annex S4: Hospitalization conditions at the Sleep Unit and applied polysomnography-montage; Annex S5: Scoring criteria applied to polysomnographic recordings; Annex S6: Description of the confounding factors included in the univariate analyses. Reference [77] is cited in the Supplementary Materials.
