# Supplementary material for: Excessive Daytime Sleepiness in Hypertensive Patients: The Role of Major Depressive Disorder
Source: Diagnostics (Basel). 2024 Aug 24;14(17):1854. doi: 10.3390/diagnostics14171854 (PMC11394253; doi:10.3390/diagnostics14171854)
Supplement: Supplementary file 1 [file diagnostics-14-01854-s001.zip › diagnostics-3159047-supplementary.pdf]

Supplementary Data

Annex S1

Outpatient care pathway for hypertensive patients

All hypertensive patients recruited for this study were initially referred to the Sleep Unit by physicians specialized in sleep medicine after an outpatient consultation during which a preliminary assessment of their complaints related to sleep, their ongoing psychotropic/somatic treatments and their somatic/psychiatric comorbidities was systematically performed to allow a first diagnostic hypothesis. Following this initial assessment, a polysomnographic recording was programmed in all these hypertensive patients to allow an objective assessment of their sleep complaints and to exclude the presence of comorbid sleep disorders that could negatively impact blood pressure control.

Annex S2

Description of blood pressure measurement by nursing staff

Systolic and diastolic blood pressures were manually measured at the right arm after five minutes of rest in a sitting position by well-trained nurses. For subjects with a systolic blood pressure ≥140 mmHg and/or a diastolic blood pressure ≥90 mmHg, blood pressures were again measured twice after a systematic rest period of five additional minutes. The first measurement was excluded whereas the second and third measurements were averaged to minimize the impact of white coat effect. In the absence of prior diagnosis of hypertension, pathological blood pressures were confirmed by repeated measurements during the stay at the Sleep Unit.

Annex S3

Description of Beck Depression Inventory reduced to 13 items, Insomnia Severity Index and Epworth Sleepiness Scale

- The Beck Depression Inventory (reduced to 13 items) was used to investigate the presence of depressive symptoms. The 13 items of this scale may be scored from 0 to 3, which means that the total score may vary from 0 to 39. A final score of 0-4 indicates an absence of depressive symptoms, 5-7 mild depressive symptoms, 8-15 moderate depressive symptoms and ≥16 severe depressive symptoms.
- The Insomnia Severity Index was used to investigate the severity of insomnia complaints. The 7 items of this index may be scored from 0 to 4, which means that the total score may vary from 0 to 28. A final score of 0-7 indicates an absence of insomnia complaints, 8-14 subclinical insomnia complaints, 15-21 moderate insomnia complaints and 22-28 severe insomnia complaints.
- The Epworth Sleepiness Scale was used to investigate daytime sleepiness. The 8 items of this scale assessing sleepiness in different daytime situations may be scored from 0 to 3, which means that the total score may vary from 0 to 24. A final score greater than 10 indicates excessive daytime sleepiness.

Annex S4

Hospitalization conditions at the Sleep Unit

The patients went to bed between 22:00 - 24:00 and got up between 6:00 - 8:00, following their usual schedule. During bedtime hours, the subjects were recumbent and the lights were turned off. Daytime naps were not permitted.

Applied polysomnography-montage

- Two electro-oculogram channels
- Three electroencephalogram channels
- One submental electromyogram channel
- Electrocardiogram
- Pressure cannula to detect the oro-nasal airflow
- Finger pulse-oximetry
- Microphone to record breathing sounds and snoring
- Plethysmographic inductive belts to measure thoracic and abdominal breathing
- Anterior tibialis electrodes

Annex S5

Scoring criteria applied to polysomnographic recordings

Obstructive apneas were scored if the decrease in air flow was ≥90% for at least 10 seconds whereas obstructive hypopneas were scored if the decrease in airflow was ≥30% for at least 10 seconds with a decrease in oxygen saturation of 3% or followed by micro-arousal. The obstructive apnea-hypopnea index corresponds to the total number of obstructive apneas and hypopneas divided by the period of sleep in hours.

Periodic limb movements were scored based on the following strict criteria: 1) duration between 0.5 to 10 seconds, 2) interval between 5 and 90 seconds from leg movement onset and 3) movements had to be part of a series of ≥4 consecutive movements meeting these criteria. Periodic limb movement index corresponds to the total number of periodic limb movements divided by period of sleep in hours.

Annex S6

Description of the confounding factors included in the univariate analyses

After a review of the literature on the risk factors for excessive daytime sleepiness [9,20,25,26,49,77], potential confounders included in this study were age (categorized: <50 years, older ≥50 years), body mass index (categorized: <25 kg/m^2, ≥25 & <30kg/m^2, ≥30 kg/m^2), dyslipidemia (categorized: no, untreated, treated), hypertension status (categorized: untreated, controlled, uncontrolled), number of antihypertensive medications (categorized: 0, 1, 2, ≥3), obstructive sleep apnea syndrome (categorized: no, mild, moderate to severe), insomnia disorders (categorized: no, sleep deprivation alone, insomnia without short sleep duration, insomnia with short sleep duration), sleep movement disorders (categorized: no, moderate to severe periodic limb movements during sleep, restless legs syndrome alone or combined with moderate to severe periodic limb movements during sleep), CRP levels (categorized: <5 mg/L, ≥5 mg/L) and as binary variables: gender, antidepressant therapy, anxiolytic medication, hypnotic medication, smoking, alcohol consumption, caffeine consumption and type 2 diabetes.
